# Creating an Inclusive Definition for High Users of Inpatient Hospital Systems That Considers Different Levels of Rurality

**DOI:** 10.3390/ijerph22030381

**Published:** 2025-03-06

**Authors:** Tomoko McGaughey, George Kephart, Utkarsh J. Dang, Paul A. Peters

**Affiliations:** 1 Department of Health Sciences, Carleton University, Ottawa, ON K1S 5B6, Canada; tomokomcgaughey@cmail.carleton.ca (T.M.); utkarshdang@cunet.carleton.ca (U.J.D.); 2 Department of Community Health and Epidemiology, Dalhousie University, Halifax, NS B3H 4R2, Canada; george.kephart@dal.ca

**Keywords:** high resource users, high system users, inpatient hospital care, survival analysis, health policy, rural health

## Abstract

Multiple definitions have been used to identify individuals who are high system users (HSUs), through economic costs, frequency of use, or length of stay for inpatient care users. However, no definition has been validated to be representative of those residing in rural communities, who face unique service accessibility. This paper identifies an HSU definition for rural Canada that is inclusive of various levels of rurality, longitudinal patient experiences, and types of hospitalizations experienced. This study utilized the 2011 Canadian Census Health and Environment Cohort (CanCHEC) linkage profile to assess hospitalization experiences between 1 January 2009 and 31 December 2013. A range of common HSU indicators were compared using Cox proportional hazards modelling for multiple periods of assessment and types of admissions. The preferred definition for rural HSUs was individuals who are in the 90th percentile of unplanned hospitalization episodes for 2 of 3 consecutive years. This approach is innovative in that it includes longitudinal hospital experiences and multiple types of hospitalizations and assesses an individual’s rurality as a point of context for analysis, rather than a characteristic. These differences provide an opportunity for community characteristic needs assessment and subsequent adjustments to policy development and resource allocation to meet each rural community’s specific needs.

## 1. Introduction

In the past, researchers have used multiple competing definitions of high system users (HSUs). These varying definitions include identifying individuals who are in a top given percentage of emergency department (ED) visits [1], frequency of ED visits [2], or frequency of family practitioner visits [3], or are in the top percentage of care costs [4]. However, these definitions are identified using an urban or combined urban/rural community sample, and do not consider the health and care service use patterns of rural communities [5].

Depending on the definition, between 15 and 20 percent of the Canadian population lives in rural areas, and this is distributed across 95% of the Canadian landmass of varying landscapes [6]. Rural Canadians face unique challenges in accessing health and care services. A smaller proportion of the rural population has a primary care physician compared to urban residents, and even fewer have access to non-ED walk-in health facilities for non-emergent care [7,8,9]. Unsurprisingly, there is a widespread lack of specialist or community care services, and the travel time to access services is much higher [10,11,12,13]. As a result, rural community residents are often left with no option but to use hospital EDs for prescription refills, non-emergency services, or physician consultations [10,12].

These unique challenges experienced in rural typologies make it difficult to use indicators that are commonly used in urban typologies, such as the number of physician visits, as these populations do not have the same level of accessibility to family physicians, or the use of frequency of ED visits when rural typologies experience limited access to community-based healthcare options [10,13,14,15,16,17]. Additionally, rural residents have trouble being discharged in a timely manner, because of a lack of referral services, home care, or transportation limitations, thus extending the length of stay [18]. This extension of the length of stay results in additional expenditure using hospital resources, but confounds an HSU definition that focuses solely on the number of hospital visits, percentile of hospital costs, or comorbidities.

The objective of this research was to explore a series of potential hospital-based HSU definitions, drawing on those used in the literature, and assess them through a standardized protocol. This includes a series of phases to identify a longitudinal frame for analysis (sensitivity analysis 1), identify types of hospitalization for assessment (sensitivity analysis 2), and finally compare definitions based on the identified parameters, to identify a rural-centric definition of HSUs in Canada. A rural-centric definition will allow for improved identification of rural HSUs, further analysis of HSUs by individual and community characteristics, and the development of policies to address health service inequities. This is of particular importance for high-income post-industrial countries such as Canada, which have ageing rural populations and often face significant resource constraints in the delivery of rural health and care services [19,20]. Identification of a rural-centric definition that is inclusive of those who live in rural spaces will enable assessment of region-specific HSUs and the development of policies and initiatives that address rural-specific needs. Addressing the health and care needs of these individuals and reducing unnecessary experiences with the healthcare system is an essential part of developing a sustainable healthcare system and nurturing a satisfactory attitude towards the public healthcare system.

## 2. Materials and Methods

### 2.1. Study Area

Canada’s vast and unique landscape provides a unique context for population health research. As the second largest country by landmass (9,194,354 square kilometers), with the world largest proportion of fresh water, touching three oceans, fifteen terrestrial ecozones, and five marine ecozones, the variety of geographies experienced in Canada is nuanced [21]. This varied landscape has not been inhabited equally, and has been molded based on physical, cultural, historical, and economic relationships over time [21]. The way in which individuals utilize their social and physical surroundings varies as well, resulting in wide variety in health behaviour practices, accessibility to healthcare resources, and other societal resources that can impact health [22,23,24]. This is similarly experienced in countries like the United States that also have varied geographies [25,26]. Given this unique intersection of geographical experiences and population distribution and their impact on health, it is important to assess health outcomes in the context of an individual’s geographical location, as the place(s) in which they are born, live, and die has a direct influence on their experience of the healthcare system.

#### 2.1.1. Provincial Administration of Healthcare in Canada

As of 31 December 2024, Canada’s total population is estimated to be 41,465,298, with the three most populous provinces being Ontario (16,171,802), Quebec (9,100,249), and British Columbia (5,719,594), while the three smallest populations are associated with the Territories (Yukon—46,948, Northwest Territories—44,936, and Nunavut—41,258), with these trends being consistent over the last 20 years [27]. As healthcare in Canada is managed by the provincial government, this makes it important to consider how the Canadian population is distributed in each region. A presentation of each province’s estimated population, as of 31 December 2024, has been provided in Table 1.

With these differences in provincial populations, it is important to use alternative geographical groupings, rather than provinces/Territories by themselves, for geographical analyses. To address this, Statistics Canada has recommended the use of Standard Geographical Classifications, to provide a systematic classification of the geographical area of Canada [28]. These regions allow for provinces or Territories that have smaller populations, but similar distribution, geographical features, and environment, to be grouped together [28]. The grouping also allows researchers to maintain statistical power in their analyses when comparing provinces, so small population counts do not limit the interpretability of results [28]. When grouped together, the small populations in the Atlantic and Prairie regions become comparable to that of Ontario, Quebec, and British Columbia.

The regionalism developed from these groupings is founded on social psychological conceptualization of these individuals as being attached to their community, institutions, and characteristics of the given geographic area [29,30]. For example, those who live in Atlantic Canada are noted to connected their identity with living on the shorelines of the Atlantic Ocean, those who live in Quebec identify with historical French culture, and those who live in British Columbia associate themselves with the surroundings of mountain ridges and temperate rainforest [31,32]. Although subgroups (i.e., Maritimes) or cross-cultural experiences (i.e., Acadian culture in Atlantic Canada) may have related cultural experiences between regions, each space has distinct geographical features [29,30,31,32]. These geographic features not only influence population distributions, as noted in the population ecumene section, but also affect the accessibility of healthcare resources, particularly for those that live in rural communities.

For this article, analysis will be conducted by the identified provincial groupings identified in Table 1. Although healthcare is provided by each provincial government, the socio-economic and geographical similarities between the provinces within each region make these groupings and this comparison analysis valid in this setting. Given that the focus of this research is rurality, not having these groupings would limit the power and interpretability of the results. The similarities within the groupings allow for reasonable interpretability and a large enough sample, stratified by rurality.

#### 2.1.2. Defining Rurality

Given the vast geographical and cultural mosaic that is Canada, the concept of what exactly are urban and rural communities varies widely. From a technical perspective, policy analysts assessing rurality typically look at the size of the rural population and what indicators they can use to support what they are exploring [33]. In contrast, social scientists, when considering rurality, often focus on fundamental dynamics of communities and the unique geographic and cultural experiences of places.

When assessing populations, it is important to consider the spaces that are being assessed. For example, are we assessing a population’s rurality based on their neighbourhood, communities, cities, regions, or provinces? Also, if the analysis is region-based, it is important to also consider differences within the region. Some define a community as rural based on the geography, regional settlement patterns and isolation, population density, commuting distances, accessibility to goods and services, and/or local economy base [33,34,35,36,37].

For example, for both the United States and the United Kingdom, if a county or county-equivalent entity (United States), or an output area (United Kingdom), has a population density of less than 10,000 people, it is considered rural [36,37]. Although a similar definition could be applied to Canada, the population density is comparatively low, given the sparsely distributed population settlement.

For Australia, the Rural Remote and Metropolitan Areas Classification (RRMA), the Accessibility/Remoteness Index of Australia (ARIA), and the Australian Standard Geographical Classification (ASGC) have been developed with the intention of measuring remoteness, taking into consideration a combination of population density and accessibility to a range of services [38,39]. All three are used in differing contexts of research and policy application, but it is agreed among researchers and policy developers that they allow an accurate understanding of the remoteness that individuals experience throughout Australia [37,38].

In Canada, there are several definitions for identifying urban and rural communities, given the diverse distribution of the population throughout the country and by province, differing based on their intended use [40]. For the purposes of this analysis, rurality was identified using the Statistics Canada Index of Remoteness (IOR), based on the population size and accessibility to goods and services by Census Subdivision (CSD), used as a proxy for rural communities [40]. For these analyses, CSDs were categorized into 5 groups, based on the natural distribution of IOR scores: Easily Accessible, Accessible, Less Accessible, Remote, and Very Remote Area [40,41].

### 2.2. Data

This analysis used the 2011 Canadian Census Health and Environment Cohort (CanCHEC) from Statistics Canada, available through the Research Data Centre program. The 2011 CanCHEC is a population-based, probabilistically linked dataset combining the population characteristics information from the 2011 National Household Survey (NHS), linked to the administrative health data and annual Postal Codes of the individuals who consented to linking their data at the time of survey completion [42]. Only those who were 18 years or older at the time of the 2011 NHS were included in this analysis.

Also linked is the Discharge Abstract Database (DAD), which includes summary clinical information for all inpatient hospitalization separations and is maintained by the Canadian Institute of Health Information (CIHI) [43]. Reported by fiscal year, data are available from 1 April 2000–31 March 2017, and provides information of the dates of admission and discharge, types of admissions, and reasons for admissions.

The CanCHEC also includes a historical Postal Code file, reporting individuals’ mailing addresses between 1986 to 2016, which showed a 93% accuracy rate for the Postal Codes provided through the T1 Family File (T1FF) form and the Census [44]. This strong agreement of presented Postal Code with actual residence allows for more accurate coding of an individual’s residential provincial region and rurality over time. Participants’ person years were excluded from the analysis if they resided outside of the study region (i.e., Quebec, territorial regions, outside of Canada). Linkage between the IOR and the reported historical Postal Code was made through the Postal Code Conversion File + (PCCF+), version 7E [45].

For this analysis, DAD records for the 2.5 years (total 5 years, 1 January 2009–31 December 2013) before and after the NHS survey date were included. A 4.5-year window (total 9 years—1 January 2007 to 31 December 2015) was used to cross-verify the results to ensure longitudinal strength, as well as analysis using the 2016 CanCHEC cohort for 2.5 years (total 5 years, 1 January 2013–31 December 2018) before and after the 2016 Census of population, which was used to verify the outputs.

All statistical analyses were performed using SAS version 9.3 (SAS Institute Inc.; Cary, NC, USA).

### 2.3. Analytic Model

The analysis’s objective was to identify the definition that is the most inclusive of those who live within rural communities, while maximizing the capture of an individual’s hospital service use and socio-demographic characteristics. The identification, assessment, and selection of the preferred indicator assumed the following: (1) that the hospital visited is community-based for those in need of inpatient care; (2) that hospitals discharge patients when they deem it appropriate; (3) that the care provided, including transfers to additional units, are necessary for patient health; and (4) that patients utilize community-based health and care resources to the best of their ability outside of the hospital.

A Cox proportional hazards model was used to evaluate different definitions for HSUs according to different degrees of rurality. Age group, region, and sex were included as class covariates, and the model was stratified by community-level IOR group. This modelling approach was selected as it can account for migration between communities over time. Events were defined as when as an individual met the required HSU definition for a given analysis. Individuals who met the assessed definition requirements in the 10 years before the analysis frame were excluded from the analysis.

The sex variable featured in the 2011 NHS and 2016 Census of Population only reports on an individual’s biological sex through binary measures (male and female), and no variable to identify the individual’s gender through these census cycles was available [46,47]. Although there are known differences in healthcare engagement behaviours between sex and gender for physical health, mental health, and health outcomes [48], these data limitations mean that this analysis cannot provide any insight into the experiences of different genders, including the experiences of those who are transgender, non-binary, or gender-diverse.

Reference groups for the modelling process were 85+ years old for age groups, British Columbia (BC) for provincial region of residence, and male for sex. Individual participants person years for analysis were excluded if they resided in Quebec, the Territories, or outside of Canada during a given study year.

### 2.4. Analytical Process

Analysis was conducted in a three-step process. First a sensitivity analysis of four-time frames was conducted to determine the time reference used to assess the definitions. Second, a comparison of types of hospitalizations was assessed to determine the effects of subgrouping hospitalization types. Third, a comparison of the identified definitions with the parameters in the first two definitions was developed.

#### 2.4.1. Model Comparison Measures

Each definition model was compared and rated based on a series of criteria: Number of Events, Regression Model Outcomes, and Applicability. For number of events, we assessed if events were distributed through the IOR groupings as expected based on population distribution within the groupings and if there were enough events to conduct further survivability analysis.

Applicability refers to the qualitative assessment of how this definition could be applied to a policy or administrative setting, including its feasibility to accurately measure in a timely way, relevance to a policy and administrative setting, and interpretability. Assessment of these criteria were coded based on the qualitative ratings of: a (good), b (moderate), and c (not suitable). These criteria were then compared to provide an overall qualitative rating, following the same scheme. We define sensitivity analysis here as a model-building step, in which model building components are explored/investigated.

#### 2.4.2. Sensitivity Analysis 1

The first round of analysis undertaken in this study evaluated the time-window for analysis to be used within a 5-year window of analysis for this definition development. The brackets of reference assessed were any instance in the analysis window, 2 of 3 years, 2 of 5 years, or 3 of 5 years. These brackets were chosen as they are representative of what has previously been used in research and are considerate of how this definition could be applied in policy in the future. All modelling conducted was based on the definition of the 90th percentile, or the top 10% of individuals with reported hospitalizations per calendar year for total hospitalizations.

#### 2.4.3. Sensitivity Analysis 2

A second analysis was conducted by the type of hospitalizations considered for HSU definitions. First, a comparison of total hospitalizations (TH) versus episodes of hospitalization (EoH) was conducted. TH enumerate each instance where an individual enters a new unit within the hospital. In contrast, EoH enumerates all transfers during a hospitalization period as a single event, including hospitalization, transfers, and readmissions within 24 h of discharge.

Secondly, unplanned, and all admissions were compared. Unplanned inpatient admissions are defined as non-elective admissions as reported through the DAD as provided by CIHI, including the patients experienced acute care portion (patient actively receiving care) and the patient alternate level of care (patient no longer requires acute care but occupies a hospital bed while awaiting placement in another healthcare facility) [49]. All admissions include unplanned and planned admissions, which are those receiving treatment for chronic conditions that cannot be administered in the community for logistical or resource limitations [49]. Assessing individuals for unplanned admissions allows for a better understanding of the characteristics for those who are experiencing preventable admissions.

#### 2.4.4. Definition Comparison

Our third and final analysis included a comparison of common definitions of HSUs, including percentile measurements, comorbidity indices, and length of stay, using the parameters identified in the earlier two analyses. The first group of indices, percentiles, identified an individual’s use of healthcare services in comparison to the rest of the study group, assessing whether they were identified to be in the upper percentile of the population group based on the given parameters. The second group of indices assessed the individual’s hospitalization experience, consisting of readmission day intervals or various interpretations of the individual’s actual length of stay over expected length of stay, based on CIHI algorithmic measurements. The final group of indices consisted of the Charlson and Elixhauser Comorbidity Indices (CCI and ECI, respectively), and assessed the types of conditions that individuals reported through the International Statistical Classification of Diseases and Related Health Problems—10th Revision (ICD-10) codes [50]. The ICD-10 classifies diseases through a system of categories to which morbidities are assigned based on established criteria. Used for both epidemiological and health management purposes, the use of ICD-10 codes allows for insight into the conditions experienced within a sample population [49].

A total of 18 indicators from 5 distinct types were assessed to identify which indicator was best suited for identifying HSUs across all rural levels.

## 3. Results

### 3.1. Analysis 1—Time-Windows

Table 2 provides the rated outcomes from sensitivity analysis 1, which compared potential analysis windows of reference for a longitudinal assessment of HSUs. The model for any instance of HSU was the strongest; however, this did not assess HSUs through a longitudinal frame, as desired in this analysis.

The 3 of 5 years model had the lowest number of events distributed across IOR groupings, potentially limiting future analysis. In addition, the lack of expected trends by age group (e.g., between the 65–84-year age group and the 85+ year age group) and sex within the model, compared to current knowledge about HSUs, was concerning. The longer period presented in this definition may also lead to delays in the identification of HSUs and action through policy or programming, limiting its applicability in a policy setting.

The 2 of 5 years model had a reasonable distribution of events across IOR groups, but there was no consistent trend in the hazard ratios and low significance of covariates, leading us to interpret the model as being ineffective in properly capturing the desired population. Also, this model runs into similar applicability issues as the 3 of 5 years model.

The preferred period for assessing HSU definitions was the 2 of 3 years model. This bracket resulted in a reasonable distribution of events across IOR groupings, had the expected hazard ratio outcome distributions, and can be easily applied in a policy setting. All results were cross-verified through an analysis using the 9-year time window of the 2011 CanCHEC model and the 5-year time window of the 2016 CanCHEC model. Supporting tables are included in Appendix A. This includes events distributed by rural groupings for each regression analysis (Table A1) and regression analysis outputs supporting the assessment of Table 2 (Table A2).

### 3.2. Analysis 2—Types of Hospitalizations

Table 2 also presents the outcomes comparing TH, EoH, any admission, and only unplanned admissions, based on the criteria outlined in Section 2.4.2.

First, this analysis compared the use of TH versus EoH. The hazard ratios for both definition types were significant and exhibited protective effects for each of the four explanatory variables. There were more events captured with better distribution across IOR groups for total hospitalizations compared to EoH (see Table A1 in Appendix A). However, when considering applicability to a policy setting, the use of episodes may better represent the HSU population. Thus, both TH and EoH have benefits and drawbacks that should be explored through the definition assessment.

The second step was to compare all types of admissions to unplanned admissions. There were no significant differences between the two regression model outputs. Although there were fewer events in the unplanned admissions models, the lack of change in the regression output leads us to believe that the model strength was not affected by these case exclusions (see Table A3 in Appendix). In terms of applicability, assessing individuals through unplanned admissions would be a more relevant representation of HSU, as this would represent potentially avoidable admissions. Thus, unplanned admissions are a preferable definition for HSUs, although both TH and EoH could be sufficient.

### 3.3. Indicator Comparison Analysis

Table 3 presents outcomes from the analysis of additional HSU indicators. First, the CCI and ECI did not rate highly for the purposes of identifying rural HSUs, as neither index identified large numbers of events overall or across IOR groups, resulting in non-converging models. Second, the 7-day readmission indicator had few events and poor regression model outcomes, capturing a small percentage of the healthcare user population. These parameter assessments also hold true for the 14-day and 30-day readmission indicators. Third, the indicators for instances of hospitalization (≥4 and ≥6) for both total hospitalizations and episodes of hospitalization have good applicability, as they are easy to measure through available records, but had too few instances of events to make characteristic analysis possible.

The strongest indicator groups were the percentile definitions and actual length of stay (ALOS)/estimated length of stay (ELOS) measurements. Models in these two indicator groups had sufficient events across IOR groups, and consistently significant regression model outcomes. ALOS/ELOS indicators have moderate applicability. Although they would be good for capturing those who use excessive resources based on their patient parameters, these indicators are dependent on third-party information from CIHI, resulting in potential analytic delays for healthcare administrative parties. Additionally, the algorithm to define ELOS is based on a nationally representative sample that could be misrepresentative of local areas.

When assessing the differences between percentile definitions, the 99th percentile for both TH and EoH unplanned admissions had insufficient events to ensure long-term sustainability of the definition and applicability in a policy setting. The 95th percentile for both admission types had more cases, but the number of events would still capture only a small proportion of the population. The 90th percentile for both TH and EoH provided enough events, maintained model strength, and would be an easy indicator to measure given the current administrative data structure, as well as being easy to interpret.

Taking into consideration the combination of all reported factors, the 90th percentile for EoH was strongest in identifying HSUs across differing IOR groups. This held true for the reported 5-year and 9-year 2011 models, and the 5-year 2016 model.

Supporting tables, including regression analysis outputs for percentile measures (Table A4), instance measures (Table A5), readmission measures (Table A6), length of stay measures (Table A7), and stratification distribution (Table A1), can be found in Appendix A.

## 4. Discussion

It is important to consider the application and implications of the research outcomes in the evaluation of indicators for researching rural HSUs. An individual’s residential rurality is often overlooked in HSU characterization; definitions that identify this factor provide an opportunity for area-specific initiatives [16,22,23,24]. This evaluation provides a guide to selecting appropriate indicators in rural Canada, and presents the relative strength based on multiple dimensions. Representation of rural communities was ensured by including measures of rurality in the assessment of HSUs, improving the potential for targeted analysis in these regions [22,23,24]. The application of this methodology will allow for an equitable understanding of those who live in rural spaces, who are otherwise overlooked. This will allow area-specific initiatives to be developed based on data of those who live in the area, rather than based on information focused on their urban counterparts.

The approach undertaken in this study developed a definition for rural HSUs using large, longitudinal linked data sources, improving on cross-sectional approaches undertaken by prior efforts. Previous studies identifying and characterizing HSUs have approached the issue through a cross-sectional or annual means, not always differentiating between the types of admissions experienced, and often assessing either only a rural community, only an urban population, or assessing rurality as a characteristic, not as a defining feature of the population, as shown in this analysis [1,2,3,4,5,10,12,13,14,15,16,17,18]. The methodology proposed and validated through this analysis minimizes identification of those who experience trauma or acute incidences and prioritizes those who receive care for chronic conditions, or who require hospitalization due to a lack of preventative, primary, or community care, which has been identified as a target area by those who provide care in rural Canada [11,12]. The identification of these rural HSUs, their characteristics, and their access to healthcare resources will facilitate an improved understanding of how resource allocation can be modified and of potential interventions to reduce high service use.

The specification of admission type in our methodology allows for the evaluation of models comparing unplanned admissions (the result of emergency or acute situations) versus planned admissions (those that are from the directive of community-based care providers). The types of hospitalizations covered in previous research are inconsistent, or not formally defined, leaving potential interpretation of the presented results [1,2,3,4,5,10,12,13,14,15,16,17,18]. This methodology encourages the specification of population underassessment, with the implications that if planned or unplanned hospitalizations are identified, tailored community-based policies or initiatives can be developed to meet that population’s needs. Future research will assess whether there is a significant difference in the socio-demographic characteristics of individuals admitted for planned versus unplanned episodes. This will allow us to better understand the use of planned healthcare services by degrees of rurality. This could also be initiated in the health administrative setting, with program or system evaluators using this definition to identify HSUs in their local communities, in a way that is inclusive of the experiences of those who live in rural spaces.

This analysis also compared indicators that have been independently used in past HSU research. Each of these indicators is appropriate for specific settings, but our methods allow for cross-comparison of each indicator in terms of applicability to research on rural HSUs.

Additionally, the strength of this analysis was in the definition of the selected rurality. The IOR allowed for assignment of rurality value based on proximity to goods and services on a continuous scale that is otherwise not captured in other definitions. Further, stratifying the analysis by custom categories allowed us to evaluate the validity of definitions for the differing degrees of rurality experienced, which was the key goal of this research.

Our analysis was significantly strengthened by utilizing a nationally sampled and representative data source linking Census characteristics to hospitalization, place of residence, and vital statistics. The results of our analysis are thus applicable for all provinces of Canada and can account for internal migration, except for Quebec and the Territories. Future analysis for the Territories will be needed to understand use of their healthcare services in a way that is representative of their accessibility, population distribution, and experiences. Analysis integrating Quebec will be conducted if the requisite hospitalization information is made available to Canadian researchers.

One limitation of this research is that it used data that are over 10 years old. Although not presented for brevity, cross-comparisons of these indicators through a 4.5-year span (9-year window) for the 2011 CanCHEC profile and a 2.5-year span (5-year window) for the 2016 CanCHEC profile were generated. Through these additional assessments, we took into consideration changes in demographic, economic, systematic, and political changes that have occurred in the Canadian healthcare system over time and how they might alter our modelling output. Given that healthcare systems are provided through provincial governance, resulting in differing care provision, the use of provincial regions as a dependent variable allowed for the control and understanding of the impact these spaces have on the definition developed. This analysis excluded periods that may have been impacted by the COVID-19 pandemic. Further assessment of this definition should include the use of the 2021 CanCHEC profile, to see if this definition holds true and identifies the same individuals over time. This analysis would further validate the strength and applicability of this definition in the context of political, systemic, socio-demographic, or economic changes. It would also show if the definitions or experiences of HSUs were impacted through the experience of an international pandemic.

In addition, by verifying the output of this model through the 2021 CanCHEC profile, this analysis will allow future insight into experiences according to gender, including the experiences of those who are gender-diverse. Although the lack of analysis by gender limits our understanding in this context, the information provided in this dataset is the best available.

It is known that differences exist across the landscape of Canada, be they based on rurality, age distribution, provincial groupings, or sex. By developing a method for identifying individuals who are using the healthcare system excessively, in a chronic way, that is considerate of these differences, this allows for an optimized opportunity to adequately identify and predict trends in healthcare service use among the Canadian population. These insights can be used in the future distribution of resources and development of policies based on population-based needs. Although the identification of this definition has been presented using data from the 2011 CanCHEC, the results have been validated using the most recent iteration that is available for analysis, the 2016 CanCHEC, which identified similar trends and results. In the present context of the Canadian healthcare system, this definition can be applied to identify and characterize HSUs based on data provision. As this analysis has been verified using a generalized, nationally representative sample, population-specific analysis can be conducted using this definition to identify and develop initiatives to address the occurrence of HSUs. This includes application to the investigation of socio-demographic characteristics by province, older adults, health behaviours, and/or condition types associated with HSU status.

Finally, those who were excluded from the original sampling frame of the CanCHEC were excluded from this analysis, including those that live in institutionalized facilities, First Nations reserves, or who are full-time members of the Canadian Forces. Future analysis could focus on HSU definitions that capture those who live in these regions. Additionally, Canadians without a fixed address may have been excluded from the analysis, as they may not have been properly captured.

## 5. Conclusions

This research has systematically developed a definition of HSUs representing individuals living across different rural settings. From this analysis, individuals from rural Canada should be considered an HSU if they are part of the 90th percentile of episodes of hospitalization experiences in 2 of 3 consecutive calendar years for unplanned admissions to hospital. The further application of bracket years and admission types adds temporality and specificity to the identification of HSUs, in a way that we believe properly identifies those who may not be receiving adequate care in the community setting. This definition provides a means by which researchers, health system planners, and policy makers can target those who are most vulnerable to becoming an HSU in rural Canada. Given the goal of equal access to care, this research is important in that it focuses explicitly on rural areas, while recognizing variation in what constitutes rurality.

## Figures and Tables

**Table 1:** Estimated population of Canada, its provinces, and its provincial regions, as of 31 December 2024.

Geography	Provincial Population as of 31 December 2024	Provincial Region	Regional Population as of 31 December 2024
Canada	41,288,599		
Newfoundland and Labrador	545,247	Atlantic	2,654,526
Prince Edward Island	178,550		
Nova Scotia	1,076,374		
New Brunswick	854,355		
Quebec	9,056,044	Quebec	9,056,044
Ontario	16,124,116	Ontario	16,124,116
Manitoba	1,494,301	Prairies	7,622,889
Saskatchewan	1,239,865		
Alberta	4,888,723		
British Columbia	5,698,430	British Columbia	5,698,430
Yukon	46,704	Territories	132,594
Northwest Territories	44,731		
Nunavut	41,159		

**Table 2:** Qualitative rating for sensitivity analysis 1 and 2.

Indicator	Number of Events	Regression Model Outcomes	Applicability	Overall Rating
Analysis 1				
Any year	a	c	c	b
2 of 3 years	a	a	b	a
2 of 5 years	a	b	b	c
3 of 5 years	b	b	a	c
Analysis 2				
Any admission (TH ^1^)	a	a	c	b
Any admission (EoH ^2^)	b	a	b	b
Unplanned admissions (TH)	a	a	b	a
Unplanned admissions (EoH)	b	a	a	a

Note: Qualitative ratings: a (good), b (moderate), and c (not suitable); ^1^ TH = total hospitalizations; ^2^ EoH = episodes of hospitalization.

**Table 3:** Qualitative rating of Indicator Comparison Analysis.

Hospitalization Indicator			Distribution of Events	Regression Outcomes	Applicability	Outcome Ranking
Percentiles	90th	TH	a	a	b	b
		EoH	a	a	a	a
	95th	TH	b	b	a	b
		EoH	b	b	a	b
	99th	TH	b	b	a	b
		EoH	b	b	a	b
Instances	≥4	TH	c	b	a	b
		EoH	c	b	a	b
	≥6	TH	c	b	a	b
		EoH	c	b	a	b
Read. ^1^	7-day	EoH	c	c	b	c
	14-day	EoH	b	b	b	b
	30-day	EoH	b	b	b	b
LOS ^2^	ALOS/ELOS > 1	TH	a	a	c	b
	ALOS-ELOS > 1	TH	a	a	c	b
	Average ALOS/ELOS > 1	TH	a	a	b	b
Ind. ^3^	CCI ≥ 5	TH	c	c	c	c
	ECI ≥ 4	TH	c	c	c	c

Note: Qualitative ratings: a (good), b (moderate), and c (not suitable); ^1^ Read. = readmission; ^2^ LOS = length of stay; ^3^ Ind = clinical indicators.

## Data Availability

The data that support the findings of this study are available from the Statistics Canada Research Data Centre, but restrictions apply to the availability of these data, which were used under license for the current study, and so are not publicly available.

## Abbreviations

The following abbreviations are used in this manuscript:

HSU	High system user
ED	Emergency department
CanCHEC	Canadian Census Health and Environment Cohort
NHS	National Household Survey
DAD	Discharge Abstract Database
CIHI	Canadian Institute of Health Information
T1FF	T1 Family Files
IOR	Index of Remoteness
CSD	Census Subdivision
PCCF+	Postal Code Conversion File +
SAS	Statistical Analysis System
BC	British Columbia
TH	Total hospitalizations
EoH	Episodes of hospitalization
CCI	Charlson Comorbidity Index
ECI	Elixhauser Comorbidity Index
ICD	International Classification of Diseases
ALOS	Actual length of stay
ELOS	Estimated length of stay
Read.	Readmission
Ind.	Clinical indicators
LOS	Length of stay
HR	Hazard ratio
CI	Confidence interval

## Appendix A

**Table A1:** Reported Distribution of Events and Censorship percentage for all regressions conducted during the study.

	IOR 1		IOR 2		IOR 3		IOR 4		IOR 5	
	Event (N)	Censor (%)	Event (N)	Censor (%)	Event (N)	Censor (%)	Event (N)	Censor (%)	Event (N)	Censor (%)
Sensitivity Analysis 1—Window of Reference Analysis										
90th percentile (TH-Any)	78,645	13.2%	43,810	14.7%	25,170	16.0%	18,395	16.2%	6740	21.2%
90th percentile (TH- 2/3 Yr^1^)	10,130	1.7%	6260	2.1%	4245	2.7%	3405	3.0%	1430	4.5%
90th percentile (TH—2/5 Yrs)	10,130	1.7%	6555	2.2%	4245	2.7%	3405	3.0%	1460	4.6%
90th percentile (TH—3/5 Yrs)	1190	0.2%	1190	0.4%	630	0.4%	570	0.5%	285	0.9%
Sensitivity Analysis 2—Types of Hospitalization Analysis										
90th percentile (TH-All)	10,130	1.7%	6260	2.1%	4245	2.7%	3405	3.0%	1430	4.5%
90th percentile (EoH-All)	10,130	1.7%	6555	2.2%	4090	2.6%	3295	2.9%	1270	4.0%
90th percentile (TH-UP^2^)	10,130	1.7%	5665	1.9%	3620	2.3%	2950	2.6%	1495	4.7%
90th percentile (EoH-UP)	7575	1.3%	4140	1.4%	2510	1.6%	2050	1.8%	860	2.7%
Indicator Comparison Analysis										
90th percentile (TH-UP)	10,130	1.7%	5665	1.9%	3620	2.3%	2950	2.6%	1495	4.7%
90th percentile (EoH-UP)	7575	1.3%	4140	1.4%	2510	1.6%	2050	1.8%	860	2.7%
95th percentile (TH-UP)	1290	0.2%	795	0.3%	530	0.3%	495	0.4%	290	0.9%
95th percentile (EoH-UP)	3185	0.5%	1805	0.6%	1115	0.7%	1050	0.9%	435	1.4%
99th percentile (TH-UP)	190	0.03%	140	0.05%	100	0.06%	95	0.09%	70	0.2%
99th percentile (EoH-UP)	430	0.07%	280	0.09%	175	0.11%	180	0.2%	85	0.3%
GE^3^ 6 Admissions (TH-UP)	145	0.02%	105	0.04%	60	0.04%	70	0.06%	60	0.2%
GE 6 Admissions (EoH-UP)	115	0.02%	75	0.03%	35	0.02%	40	0.03%	25	0.08%
GE 4 Admissions (TH-UP)	770	0.1%	530	0.2%	355	0.2%	380	0.3%	235	0.7%
GE 4 Admissions (EoH-UP)	620	0.1%	390	0.1%	240	0.2%	235	0.2%	110	0.4%
ALOS/ELOS > 1 (TH-UP)	29,510	4.9%	16,760	5.6%	9940	6.3%	7105	6.2%	2300	7.2%
ALOS-ELOS > 1 (TH-UP)	21,825	3.6%	12,405	4.1%	7430	4.5%	5185	4.5%	1550	4.9%
Avg ALOS/ELOS > 1 (TH-UP)	18,030	3.0%	9625	3.2%	5545	3.5%	3885	3.4%	1120	3.5%
7-day Read. (EoH-UP)	1515	0.3%	765	0.3%	435	0.3%	380	0.4%	140	0.5%
14-day Read. (EoH-UP)	3645	0.7%	1840	0.7%	1125	0.8%	855	0.8%	325	1.1%
30-day Read. (EoH-UP)	7175	1.3%	3660	1.3%	2140	1.5%	1675	1.6%	595	2.0%

^1^Yrs = years, ^2^UP = unplanned, ^3^GE = greater than or equal to.

**Table A2:** Analysis of maximum likelihood estimates for sensitivity analysis 1.

	90th Percentile (Any Instance—All)		90th Percentile (2 of 3 Years—All)		90th Percentile (2 of 5 Years—All)		90th Percentile (3 of 5 Years—All)	
Parameter	*p* Value	HR (95% CI)	*p* Value	HR (95% CI)	*p* Value	HR (95% CI)	*p* Value	HR (95% CI)
18–44	<0.0001	0.443 (0.423, 0.452)	<0.0001	0.244 (0.232, 0.256)	<0.0001	0.244 (0.232, 0.257)	<0.0001	0.31 (0.272, 0.352)
45–64	<0.0001	0.692 (0.678, 0.705)	<0.0001	0.487 (0.466, 0.510)	<0.0001	0.486 (0.464, 0.508)	<0.0001	0.603 (0.536, 0.678)
65–84	<0.0001	0.958 (0.939, 0.976)	<0.0001	0.839 (0.803, 0.877)	<0.0001	0.836 (0.800, 0.873)	0.1787	0.925 (0.825, 1.036)
Atlantic	<0.0001	0.738 (0.728, 0.749)	<0.0001	0.619 (0.597, 0.642)	<0.0001	0.622 (0.600, 0.645)	<0.0001	0.55 (0.503, 0.601)
Ontario	<0.0001	0.692 (0.684, 0.700)	<0.0001	0.546 (0.529, 0.564)	<0.0001	0.543 (0.527, 0.561)	<0.0001	0.403 (0.371, 0.439)
Prairies	<0.0001	0.778 (0.770, 0.787)	<0.0001	0.761 (0.740, 0.782)	<0.0001	0.758 (0.738, 0.780)	<0.0001	0.717 (0.670, 0.767)
Female	<0.0001	0.892 (0.884, 0.899)	<0.0001	0.814 (0.796, 0.832)	<0.0001	0.815 (0.797, 0.834)	<0.0001	0.753 (0.712, 0.796)

**Table A3:** Analysis of maximum likelihood estimates for sensitivity analysis 2.

	90th Percentile (2 of 3 Years—TH—All)		90th Percentile (2 of 3 Years—EoH—All)		90th Percentile (2 of 3 Years—TH—UP)		90th Percentile (2 of 3 Years—EoH—UP)	
Parameter	*p* Value	HR (95% CI)	*p* Value	HR (95% CI)	*p* Value	HR (95% CI)	*p* Value	HR (95% CI)
18–44	<0.0001	0.244 (0.232, 0.256)	<0.0001	0.244 (0.232, 0.256)	<0.0001	0.936 (0.928, 0.944)	<0.0001	1.079 (1.071, 1.088)
45–64	<0.0001	0.487 (0.466, 0.510)	<0.0001	0.487 (0.466, 0.510)	<0.0001	0.93 (0.923, 0.938)	<0.0001	1.046 (1.037, 1.054)
65–84	<0.0001	0.839 (0.803, 0.877)	<0.0001	0.839 (0.803, 0.877)	<0.0001	0.913 (0.906, 0.920)	<0.0001	0.98 (0.973, 0.988)
Atlantic	<0.0001	0.619 (0.597, 0.642)	<0.0001	0.619 (0.597, 0.642)	<0.0001	0.968 (0.961, 0.974)	<0.0001	0.974 (0.967, 0.981)
Ontario	<0.0001	0.546 (0.529, 0.564)	<0.0001	0.546 (0.529, 0.564)	<0.0001	0.979 (0.974, 0.984)	<0.0001	0.978 (0.973, 0.984)
Prairies	<0.0001	0.761 (0.740, 0.782)	<0.0001	0.761 (0.740, 0.782)	0.0107	0.993 (0.988, 0.998)	0.4206	0.998 (0.992, 1.003)
Female	<0.0001	0.814 (0.796, 0.832)	<0.0001	0.814 (0.796, 0.832)	<0.0001	0.981 (0.977, 0.985)	<0.0001	0.991 (0.987, 0.994)

**Table A4:** Analysis of maximum likelihood estimates for indicator comparison (percentiles).

	95th Percentile (2 of 3 Years—TH—UP)		95th Percentile (2 of 3 Years—EoH—UP)		99th Percentile (2 of 3 Years—TH—UP)		99th Percentile (2 of 3 Years—EoH—UP)	
Parameter	*p* Value	HR (95% CI)	*p* Value	HR (95% CI)	*p* Value	HR (95% CI)	*p* Value	HR (95% CI)
18–44	<0.0001	0.946 (0.938, 0.953)	<0.0001	0.947 (0.939, 0.954)	<0.0001	0.939 (0.931, 0.946)	<0.0001	1.079 (1.071, 1.088)
45–64	<0.0001	0.934 (0.927, 0.942)	<0.0001	0.937 (0.929, 0.944)	<0.0001	0.929 (0.922, 0.937)	<0.0001	1.046 (1.037, 1.054)
65–84	<0.0001	0.913 (0.905, 0.920)	<0.0001	0.914 (0.907, 0.922)	<0.0001	0.911 (0.904, 0.918)	<0.0001	0.98 (0.973, 0.988)
Atlantic	<0.0001	0.973 (0.966, 0.980)	<0.0001	0.975 (0.968, 0.982)	<0.0001	0.97 (0.963, 0.977)	<0.0001	0.974 (0.967, 0.981)
Ontario	<0.0001	0.981 (0.975, 0.986)	<0.0001	0.982 (0.977, 0.987)	<0.0001	0.98 (0.975, 0.985)	<0.0001	0.978 (0.973, 0.984)
Prairies	0.0011	0.991 (0.986, 0.996)	0.2109	0.997 (0.991, 1.002)	<0.0001	0.989 (0.983, 0.994)	0.4206	0.998 (0.992, 1.003)
Female	<0.0001	0.981 (0.978, 0.985)	<0.0001	0.981 (0.977, 0.985)	<0.0001	0.98 (0.977, 0.984)	<0.0001	0.991 (0.987, 0.994)

**Table A5:** Analysis of maximum likelihood estimates for indicator comparison (instances).

	6 or More Admissions (2 of 3 Years—TH—UP)		6 or More Admissions (2 of 3 Years—EoH—UP)		4 or More Admissions (2 of 3 Years—TH—UP)		4 or More Admissions (2 of 3 Years—EoH—UP)	
Parameter	*p* Value	HR (95% CI)	*p* Value	HR (95% CI)	*p* Value	HR (95% CI)	*p* Value	HR (95% CI)
18–44	<0.0001	0.939 (0.931, 0.946)	<0.0001	0.938 (0.931, 0.946)	<0.0001	0.941 (0.933, 0.949)	0.0051	0.692 (0.535, 0.895)
45–64	<0.0001	0.929 (0.922, 0.937)	<0.0001	0.929 (0.922, 0.936)	<0.0001	0.931 (0.923, 0.938)	0.511	1.085 (0.85, 1.385)
65–84	<0.0001	0.911 (0.904, 0.918)	<0.0001	0.911 (0.904, 0.918)	<0.0001	0.911 (0.904, 0.918)	0.8565	1.023 (0.801, 1.306)
Atlantic	<0.0001	0.969 (0.962, 0.976)	<0.0001	0.969 (0.962, 0.976)	<0.0001	0.971 (0.964, 0.978)	<0.0001	0.665 (0.544, 0.813)
Ontario	<0.0001	0.98 (0.975, 0.985)	<0.0001	0.98 (0.975, 0.985)	<0.0001	0.982 (0.976, 0.987)	0.7617	0.976 (0.835, 1.141)
Prairies	<0.0001	0.988 (0.983, 0.993)	<0.0001	0.988 (0.983, 0.993)	0.0007	0.991 (0.985, 0.996)	0.3355	0.93 (0.801, 1.078)
Female	<0.0001	0.98 (0.977, 0.984)	<0.0001	0.98 (0.977, 0.984)	<0.0001	0.98 (0.977, 0.984)	0.0263	0.882 (0.79, 0.985)

**Table A6:** Analysis of maximum likelihood estimates for indicator comparison (readmissions).

	7-Day Readmissions (2 of 3 Years—EoH—UP)		14-Day Readmissions (2 of 3 Years—EoH—UP)		30-Day Readmissions (2 of 3 Years—EoH—UP)	
Parameter	*p* Value	HR (95% CI)	*p* Value	HR (95% CI)	*p* Value	HR (95% CI)
18–44	<0.0001	0.936 (0.929, 0.944)	<0.0001	0.943 (0.935, 0.951)	<0.0001	0.955 (0.947, 0.963)
45–64	<0.0001	0.928 (0.921, 0.936)	<0.0001	0.934 (0.927, 0.942)	<0.0001	0.944 (0.936, 0.952)
65–84	<0.0001	0.911 (0.903, 0.918)	<0.0001	0.913 (0.906, 0.921)	<0.0001	0.917 (0.910, 0.925)
Atlantic	<0.0001	0.971 (0.964, 0.978)	<0.0001	0.973 (0.966, 0.981)	<0.0001	0.979 (0.972, 0.986)
Ontario	<0.0001	0.98 (0.975, 0.986)	<0.0001	0.981 (0.976, 0.987)	<0.0001	0.984 (0.978, 0.989)
Prairies	0.0006	0.99 (0.985, 0.996)	0.0441	0.994 (0.989, 1.000)	0.4833	1.002 (0.996, 1.008)
Female	<0.0001	0.98 (0.976, 0.983)	<0.0001	0.981 (0.977, 0.985)	<0.0001	0.981 (0.977, 0.985)

**Table A7:** Analysis of maximum likelihood estimates for indicator comparison (length of stay).

	ALOS/ELOS > 1 (2 of 3 Years—TH—UP)		ALOS-ELOS > 1 (2 of 3 Years—TH—UP)		Average ALOS/ELOS > 1 (2 of 3 Years—TH—UP)	
Parameter	*p* Value	HR (95% CI)	*p* Value	HR (95% CI)	*p* Value	HR (95% CI)
18–44	<0.0001	0.941 (0.934, 0.949)	<0.0001	1.035 (1.026, 1.044)	<0.0001	1.031 (1.023, 1.040)
45–64	<0.0001	0.931 (0.923, 0.938)	<0.0001	1.019 (1.011, 1.028)	0.0006	1.015 (1.006, 1.023)
65–84	<0.0001	0.912 (0.905, 0.919)	<0.0001	0.969 (0.961, 0.977)	<0.0001	0.966 (0.958, 0.975)
Atlantic	<0.0001	0.97 (0.964, 0.977)	0.0506	0.993 (0.986, 1.000)	0.0015	0.988 (0.981, 0.996)
Ontario	<0.0001	0.981 (0.976, 0.986)	0.1022	0.995 (0.990, 1.001)	0.1766	0.996 (0.991, 1.002)
Prairies	0.0002	0.99 (0.985, 0.995)	<0.0001	1.023 (1.017, 1.028)	<0.0001	1.016 (1.010, 1.022)
Female	<0.0001	0.98 (0.976, 0.984)	<0.0001	0.983 (0.979, 0.986)	<0.0001	0.982 (0.979, 0.986)
